# Frequency and Distribution of Rickettsiae, Borreliae, and Ehrlichiae Detected in Human-Parasitizing Ticks, Texas, USA

**DOI:** 10.3201/eid2202.150469

**Published:** 2016-02

**Authors:** Elizabeth A. Mitchell, Phillip C. Williamson, Peggy M. Billingsley, Janel P. Seals, Erin E. Ferguson, Michael S. Allen

**Affiliations:** University of North Texas Health Science Center, Fort Worth, Texas, USA (E.A. Mitchell, P.C. Williamson, P.M. Billingsley, J.P. Seals, E.E. Ferguson, M.S. Allen);; Creative Testing Solutions, Tempe, Arizona, USA (P.C. Williamson);; University of Utah, Salt Lake City, Utah, USA (P.M. Billingsley)

**Keywords:** zoonoses, tick, tickborne, vector, vector-borne infections, Rickettsia, Borrelia, Ehrlichia, Amblyomma, Dermacentor, Rickettsia, Ixodes, Rhipicephalus, Amblyomma americanum, Dermacentor variabilis, Rhipicephalus sanguineus, Ixodes scapularis, Amblyomma maculatum, Amblyomma cajennense, Texas, bacteria

## Abstract

To describe the presence and distribution of tickborne bacteria and their vectors in Texas, USA, we screened ticks collected from humans during 2008–2014 for *Rickettsia*, *Borrelia*, and *Ehrlichia* spp. Thirteen tick species were identified, and 23% of ticks carried bacterial DNA from at least 1 of the 3 genera tested.

Ticks are vectors for a variety of microorganisms, many of which are known agents of zoonotic disease. Although much current research is focused on areas where these diseases are common, it is crucial to collect data from areas with fewer diagnoses of tickborne illness. In Texas, USA, tickborne diseases caused by *Rickettsia*, *Borrelia*, and *Ehrlichia* bacteria are diagnosed less frequently than in some areas of the United States (*1*); however, those agents have been documented to occur (*2*), and many medically relevant tick species, capable of carrying and transmitting these pathogens, are established in various geographic areas of Texas (*1*). Long-term surveillance data encompassing consecutive seasons and a wide geographic range are necessary to ascertain disease transmission risks associated temporally or geographically with established or emerging tickborne pathogens and their vectors. The University of North Texas Health Science Center Tick-Borne Disease Research Laboratory (UNTHSC-TBDL), the primary tick-testing facility for Texas Department of State Health Services Zoonosis Control (TX DSHS), receives ticks continually throughout the year. The data collected from this testing provide an assessment of the prevalence of tick species and associated tickborne bacterial agents collected in Texas.

## The Study

From October 1, 2008, through September 30, 2014, ticks removed from humans were sent by TX DSHS to UNTHSC-TBDL, where they were tested by using PCR-based methods, then underwent by DNA sequence analysis to determine the presence of *Rickettsia*, *Borrelia*, and *Ehrlichia* spp. Morphologic identification of tick species was implemented by entomologists at TX DSHS. Ticks that could not be classified morphologically were identified at UNTHSC-TBDL by sequencing mitochondrial 16S rDNA (data not shown).

Each tick was sent to UNTHSC-TBDL in an individual collection tube. Upon arrival, ticks were processed according to the laboratory’s standard protocol, as described by Williamson et al. (*2*). After bead pulverization, we extracted DNA using the E.Z.N.A. Mollusc DNA Isolation Kit (Omega Bio-Tek, Norcross, GA, USA) following the manufacturer’s protocol.

DNA from each specimen was screened in duplicate by PCR for *Rickettsia*, *Borrelia,* and *Ehrlichia* spp. as previously described (*2*) by using primers listed in Table 1. PCR products were evaluated, and presumptive-positive amplicons were purified for sequencing (*2*). Cycle sequencing reactions were performed in both directions by using BigDye Terminator version 3.1 chemistry (Life Technologies, Carlsbad, CA, USA). Dideoxy chain termination products were detected electrophoretically on an ABI 310 or 3130xL Genetic Analyzer (Life Technologies). Sequence analysis was performed by using Sequencher version 4.8/5.0 (GeneCodes, Ann Arbor, MI, USA). Analyzed sequences were compared with reference data in GenBank (http://blast.ncbi.nlm.nih.gov/). Sequences were submitted to GenBank under accession nos. KP861333–KP861347.

**Table 1:** Primers used for screening of human-parasitizing tick specimens, Texas, USA, October 1, 2008–September 30, 2014

Primer name	Gene	Primer sequence, 5′ → 3′	Specificity	Amplicon, bp	Reference
*Borrelia* spp.					
FlaLL	*flaB*	ACATATTCAGATGCAGACAGAGGT	Genus	664	(*3*)
FlaRL	*flaB*	GCAATCATAGCCATTGCAGATTGT	Genus		(*3*)
FlaLS	*flaB*	AACAGCTGAAGAGCTTGGAATG	Genus	330	(*3*)
FlaRS	*flaB*	CTTTGATCACTTATCATTCTAATAGC	Genus		(*3*)
BL-Fla 522F	*flaB*	GGTACATATTCAGATGCAGACAGAGGG	*B. lonestari*	660	(*2*)
BL-Fla 1182R	*flaB*	GCACTTGATTTGCTTGTGCAATCATAGCC	*B. lonestari*		(*2*)
BL-Fla 662F	*flaB*	CTGAAGAGCTTGGAATGCAACCTGC	*B. lonestari*	198	(*2*)
BL-Fla 860R	*flaB*	GAGCTAATCCCACCTTGAGCTGG	*B. lonestari*		(*2*)
BL-16S 227F	16S	TCACACTGGAACTGAGATACGGTCC	Genus	693	(*2*)
BL-16S 920R	16S	GAATTAAACCACATGCTCCACCGC	Genus		(*2*)
*Rickettsia* spp.					
Rr.190 70P	* rompA*	ATGGCGAATATTTCTCCAAAA	Genus	532	(*4*)
Rr.190 602N	* rompA*	AGTGCAGCATTCGCTCCCCCT	Genus		(*4*)
BG1–21	*rompB*	GGCAATTAATATCGCTGACGG	Genus	650	(*5*)
BG2–20	*rompB*	GCATCTGCACTAGCACTTTC	Genus		(*5*)
*Ehrlichia* spp.					
Ehr DSB 330F	*dsb*	GATGATGTCTGAAGATATGAAACAAAT	Genus	398	(*6*)
Ehr DSB 728R	*dsb*	CTGCTCGTCTATTTTACTTCTTAAAGT	Genus		(*6*)
Ehr map1F	* map*1	ATTTTTACCTGGTGTGTCCTTTTCTGA	Genus	873	(*7*)
Ehr map1R	* map*1	CCTTCCTCCAATTTCTATACC	Genus		(*7*)
Ehr Pmap2F	* map*1	GACACCAAGGCAGTATACGG	Genus		(*7*)
Ehr Pmap2R	* map*1	CTAAGTCAGTACCAATACCTGCAC	Genus		(*7*)
Tick DNA					
16S-1	mt16S	CCGGTCTGAACTCAGATCAAG	Unknown	300	(*8*)
16S+2	mt16S	TTGGGCAAGAAGACCCTATGAA	Unknown		(*8*)

The TX DSHS submitted 1,112 ticks to UNTHSC-TBDL during October 1, 2008–September 30, 2014, of which 1,062 originated in Texas. Thirteen tick species were identified; most were *Amblyomma americanum* (55.7%), followed by *Dermacentor variabilis* (15.0%), *Rhipicephalus sanguineus* (13.0%), *Ixodes scapularis* (5.6%), *A. maculatum* (5.4%), and *A. cajennense* (2.9%). Approximately 23.3% of ticks originating in Texas tested positive for DNA from *Rickettsia*, *Borrelia*, or *Ehrlichia* bacteria (Table 2; Technical Appendix Table). Of these bacteria, most belonged to spotted fever group rickettsiae (SFGR); *A. americanum* was the most common tick species found to carry an SFGR agent. The most frequent SFGR sequences detected demonstrated 100% identity to *Candidatus* Rickettsia amblyommii *rompA* (GenBank accession no. EF194096). *Candidatus* R. amblyommii was detected in both *A. americanum* and *A. cajennense* ticks and showed prevalence rates of 30.3% and 32.3%, respectively. The second most common SFGR *rompA* sequences were 100% homologous to the previously termed rickettsial *I. scapularis* endosymbiont, which has been officially named *R. buchneri* (accession no. KP172259) (*9*). Five *A. maculatum* specimens contained DNA sequences identical to *R. parkeri* *rompA* (accession no. KC003476). Sequences that shared 100% similarity to 1 specific *R. rhipicephali* isolate (accession no. U43803) and 99% similarity to other *R. rhipicephali* *rompA* isolates (accession nos. EU109175–EU109178) were obtained from 4 *D. variabilis* ticks. Sequences isolated from 2 *D. andersoni* ticks were identical to *R. peacockii rompA* and *rompB* (accession nos. FM883671 and CP001227, respectively). Tick species was confirmed by sequencing mitochondrial 16S rDNA. Sequences from both specimens aligned 99% with *D. andersoni* (accession no. EU711343) and 94% with *D. variabilis* (accession no. L34300). *D. andersoni* is not known to inhabit Texas (*1*,*10*), so this finding could suggest a novel geographic association.

**Table 2:** Number of positive bacterial DNA sequences identified for each human-parasitizing tick species, Texas, USA, October 1, 2008–September 30, 2014*

Tick	No. positive								
	*Borrelia*			*Ehrlichia * *chaffeensis*	*Rickettsia *				
	UNID	*burgdorferi*	*lonestari*		amblyommii†	*parkeri*	*peacockii*	*rhipicephali*	*buchneri*
*Amblyomma americanum*	0	0	8	2	179	0	0	0	0
*A. cajennense*	0	0	0	0	10	0	0	0	0
*A. maculatum*	2	0	0	0	0	5	0	0	0
*Dermacentor variabilis*	1	0	0	0	0	0	0	4	0
*D. andersoni*	0	0	0	0	0	0	2	0	0
*Ixodes scapularis*	0	1	0	0	0	0	0	0	44
*Rhipicephalus sanguineus*	0	0	0	0	0	0	0	0	0
Total	3	1	8	2	189	5	2	4	44
*Only tick species originating in Texas that tested positive for *Borrelia*, *Ehrlichia*, or *Rickettsia* spp. by DNA sequence analysis are shown. Additionally, 2 *A*. *maculatum* ticks from Texas were positive for Panola Mountain *Ehrlichia*. UNID, unidentified species. †*Candidatus* species.									

The total prevalence of borreliae detected was 1.1%. DNA sequences sharing 100% identity to *B. lonestari* were found in 8 *A. americanum* ticks (1.4%). As seen by Stromdahl et al., the *B. lonestari* isolates matching sequences in this study depended on the insertion or deletion of a nucleotide triplet, AAG (*11*). Sequences from 7 tick samples matched 100% with *B*. *lonestari flaB* isolates containing the additional triplet (accession no. AY850063), and 1 sequence was identical to *B. lonestari flaB* isolates lacking the triplet (accession no. AY850064). Of the 8 *A. americanum* ticks from which the *B. lonestari* sequences were obtained, 6 were co-infected with *Candidatus* R. amblyommii. DNA extracts from 1 *I. scapularis* tick contained a sequence consistent with *B. burgdorferi* sensu stricto (s.s.) and was co-infected with *R. buchneri*. The *flaB* sequence matched 100% to (accession no. CP002228), and 99% to (accession no. CP009656) *B. burgdorferi* s.s. reference sequences. The *Borrelia* 16S rDNA sequence showed 100% identity to (accession no. CP009656) and differed by 1 single nucleotide polymorphism from (accession no. CP002228) *B. burgdorferi* s.s. reference sequences. A *flaB* gene sequence from 1 *D. variabilis* tick shared 100% identity with *Candidatus* B. texasensis (accession no. AF264901). Samples from 2 *A. maculatum* ticks showed *flaB* sequences matching 90% identity values to *B. turcica* (accession no. AB109243), a reptilian *Borrelia* sp. Those *flaB* sequences were identical to a novel *Borrelia* sp. (accession no. KF395230) previously found in *A. maculatum* ticks in Mississippi and known to share a phylogenetic clade with *B. turcica* (*12*). *Borrelia* 16S rDNA primers produced nonspecific amplification with these 2 samples. 

Phylogenetic analysis was performed by using MEGA version 5.1 (http://www.megasoftware.net) using GenBank reference sequences to examine relationships between the *Borrelia* sp. from this study, *B. turcica*, and both Lyme disease–associated and relapsing fever borreliae (Figure). The results supported findings by Lee et al. that the novel *Borrelia* sp. *flaB* sequences were more closely related to the reptilian *Borrelia* than the other 2 *Borrelia* groups (*12*).

**Figure:**
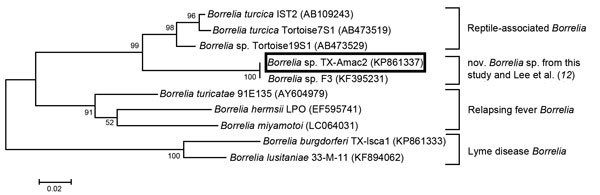
Maximum-likelihood tree showing that the novel *Borrelia* sp. identified in *Amblyomma maculatum* ticks from Texas in this study (box) and from Mississippi (*12*) shares a closer phylogenetic relationship to *B. turcica* than to to other *Borreliae* groups. Analysis is based on *flaB* sequences (267 bp). GenBank accession numbers are shown in parentheses. Tree was constructed using the Tamura 3-parameter model with a bootstrap value of 1,000 replicates. Scale bar indicates substitutions per nucleotide position.

Two *A. americanum* ticks contained DNA sharing 100% identity with *Ehrlichia chaffeensis* *dsb* (accession no. CP000236). One of these ticks was co-infected with *Candidatus* R. amblyommii. Prevalence of *E. chaffeensis* in the *A. americanum* specimens tested was 0.34%. In addition, 2 of 42 *A. maculatum* ticks tested for the emerging pathogen Panola Mountain *Ehrlichia* sp. (PME) (*7*) each produced a *map*1 sequence that was 100% homologous to 2 separate PME reference sequences (accession nos. EU272356, EU272358). These sequences differed from each other by 1 single nucleotide polymorphism. This finding represents a novel association, as *A. americanum* is the known vector for PME (*7*). A subset of 141 *A. americanum* ticks was also tested for PME, with negative results.

## Conclusions

Frequency of tickborne zoonoses in Texas remains low compared with some regions of the United States. We report the detection of known pathogens along with bacteria of unknown pathogenicity in human-parasitizing ticks commonly found in Texas. Our findings underscore the importance of better characterization and continued surveillance of the frequency and distribution of tick species and the bacterial agents they carry. Continued monitoring in low-risk areas provides data regarding the presence of potential emerging pathogens and vectors not yet commonly identified, which could pose unidentified threats to public health.

Technical Appendix Summary of number, identity, and bacterial screening results for ticks collected in Texas, USA, October 2008–September 2014.
