# Supplementary material for: Frequency and Distribution of Rickettsiae, Borreliae, and Ehrlichiae Detected in Human-Parasitizing Ticks, Texas, USA
Source: Emerg Infect Dis. 2016 Feb;22(2):312–5. doi: 10.3201/eid2202.150469 (PMC4734531; doi:10.3201/eid2202.150469)
Supplement: Technical Appendix — Summary of number, identity, and bacterial screening results for ticks collected in Texas, USA, October 2008–September 2014. [file 15-0469-Techapp-s1.pdf]

Article DOI: http://dx.doi.org/10.3201/eid2202/150469

Frequency and Distribution of Rickettsiae, Borreliae, and Ehrlichiae Detected in Human-Parasitizing Ticks, Texas, USA

Technical Appendix

Technical Appendix Table. Summary of number, identity, and bacterial screening results for ticks submitted by Texas Department of State Health Services Zoonosis Control, October 1, 2008–September 30, 2014*

	No. positive/no. tested			
Tick	*Borrelia* spp.	*Ehrlichia* spp.	*Rickettsia* spp.	Total
Amblyomma americanum				
Adult female	6/209	1/209	71/209	78/209
Adult male	1/155	1/155	41/155	43/155
Nymph	1/219	0/219	54/219	55/219
Larva	0/8	0/8	0/8	0/8
Amblyomma cajennense				
Adult female	0/5	0/5	1/5	1/5
Adult male	0/6	0/6	0/6	0/6
Nymph	0/19	0/19	9/19	9/19
Larva	0/1	0/1	0/1	0/1
Amblyomma maculatum				
Adult female	1/22	0/22	2/22	3/22
Adult male	1/29	2/29	2/29	5/29
Nymph	0/6	0/6	0/6	0/6
Larva	0/0	0/0	0/0	0/0
Dermacentor variabilis				
Adult female	1/90	0/90	2/90	3/90
Adult male	0/63	0/63	2/63	2/63
Nymph	0/4	0/4	0/4	0/4
Larva	0/0	0/0	0/0	0/0
Dermacentor andersoni				
Adult female	0/2	0/0	2/2	2/2
Adult male	0/0	0/0	0/0	0/0
Nymph	0/0	0/0	0/0	0/0
Larva	0/0	0/0	0/0	0/0
Ixodes scapularis				
Adult female	1/52	0/52	42/52	43/52
Adult male	0/1	0/1	0/1	0/1
Nymph	0/6	0/6	3/6	3/6
Larva	0/0	0/0	0/0	0/0
Total	12/897	4/897	231/897	247/897

*Table only includes ticks originating in Texas that tested positive for *Borrelia*, *Ehrlichia*, or *Rickettsia* spp. One hundred thirty-seven *Rhipicephalus sanguineus*, 12 unidentified *Amblyomma* spp., 6 unidentified *Ixodes* spp., 3 *Otobius megnini*, 2 unidentifiable ticks and 1 specimen each of *Amblyomma imitator*, *Amblyomma inornatum*, *Dermacentor albipictus*, *Dermacentor nigrolineatus*, and *Ixodes woodi* were additionally submitted from Texas.
